# Discovery of *Cinchona* as Antimalarial, Viceroyalty of Peru, Circa 1630

**DOI:** 10.3201/eid3207.260042

**Published:** 2026-07

**Authors:** Jesús Rojas-Jaimes, Stephanie Rodríguez-Gómez, Guido P. Lombardi

**Affiliations:** Universidad Privada del Norte, Lima, Peru (J. Rojas-Jaimes); Universidad Científica del Sur, Lima (J. Rojas-Jaimes); Universidad Continental Postgraduate School, Lima (J. Rojas-Jaimes); Universidad Nacional Federico Villarreal, Lima (S. Rodríguez-Gómez); Universidad Peruana Cayetano Heredia, Lima (G.P. Lombardi)

**Keywords:** cinchona, antimalarial, malaria, parasites, Peru

## Abstract

The empirical discovery of the therapeutic power of *Cinchona* tree bark in the 17th Century has been one of the most important achievements in the history of medicine in its fight against malaria. Only after 2 centuries, since the isolation of its main alkaloid, quinine, could other important antimalarials, such as chloroquine, be synthesized, all of which helped to save hundreds of millions of lives. In this historical review, we examine the evidence, accessed from early documentary sources, concerning the discovery of *Cinchona* and its therapeutic value as an antimalarial during the Viceroyalty of Peru.

The genus *Cinchona*, family Rubiaceae, comprises 23 tree species (*1*). Together, they are called *quina*, 15-meter-high trees native to South America whose bark, branches, and leaves hold an intense bitter taste (*2*). Authors such as Espinosa and Cobo have confused the *quina* trees of the *Cinchona* genus with the *quina-quina* tree of the genus *Myroxylon* (*3*).

*Cinchona*’s propensity to treat malaria came at an opportune time, when much of the population of southern Europe was experiencing this disease, during the 17th Century. Classified in 1742 by Carl Linnaeus, *Cinchona*’s processed bark was known as Peruvian, Jesuits’, countess’, Loja’s, cardinal’s, or Lugo’s powders and also as Peruvian antitertian and bark of fevers (*1*).

*Cinchona* bark was discovered in the 1600s in the Viceroyalty of Peru as a treatment that could be used to treat fevers in general. In 1820, Pierre-Joseph Pelletier and Joseph Bienaimé Caventou managed to isolate 2 alkaloids in the *Cinchona* bark, to which they attributed the febrifuge and antiparasitic properties of the substance, calling them quinine and cinconine (*4*). However, quinine is the main active principle and alkaloid in *Cinchona* for treating malaria.

Later, in 1889, Charles Louis Alphonse Laveran (winner of the Nobel Prize in Physiology or Medicine in 1907) discovered the *Plasmodium* parasite, the causative agent of malaria, and in 1897, Ronald Ross discovered the *Anopheles* mosquito, the vector that transmits *Plasmodium*. In 1902, Robert Koch implemented massive chemoprophylaxis in New Guinea, emphasizing malaria-control measures, which were effectively used in World War I. Until then, treatment of malaria relied strongly on extracts from the bark of the *Cinchona* tree for their antimalarial effect. Not until 1944, with the discovery of quinine’s molecular structure, could antimalarial drugs be synthesized on a commercial scale (*4*–*6*).

Although the initial discovery of *Cinchona*’s antimalarial effect in humans was empirical, the mechanism of action of the molecules responsible for this effect, such as the alkaloids involved in the schizonticidal effect, was subsequently elucidated. The molecules interfere with the parasite’s ability to detoxify by using quinoline. In vitro studies have been fundamental in determining those mechanisms of action. The effect of quinine inhibiting the heme polymerase extracted from *P. falciparum* trophozoites and the mechanisms of action of specific alkaloids depend on the chemical structures of quinine, quinidine, 9-epiquinine, and 9-epiquinidine mediated by the geometry of the 9-hydroxyl group and the quinuclidine ring system, which are fundamentally determined by the hydroxyl and amino groups (*7*,*8*).

Unfortunately, *Cinchona*, popularly known in Spanish as *cascarilla*, was intensively exploited after its discovery as an antimalarial, which made the trees extremely vulnerable. In addition, the tree’s trunk is not wide or robust, making it difficult to climb and therefore easy to fell with machetes, leading to trees being cut down to extract the precious *cascarilla* (*1*). Therefore, it is important to consider the harvesting, acquisition, and management of this valuable tree.

The beginning of the *Cinchona* story is less than clear. As Pratik Chakrabarti indicates, that uncertainty could have been driven by colonialist intellectual and material interests (*4*).

## Discovery of *Cinchona*’s Value as an Antimalarial

In terms of who discovered *Cinchona*’s value as an antimalarial, 2 basic assumptions exist: the natives discovered both its febrifuge and antimalarial properties consciously, and Jesuits discovered its antimalarial value after learning of its febrifuge use by the natives. Jesuit priest Sánchez Labrador tells how Loja natives learned about *Cinchona*’s value: they noticed that a man got cured of an intense fever after drinking from a bitter lake. The water had taken the flavor of fallen cinchona trees. To pinpoint the source of the cure, they soaked different tree parts, concluding that the medicinal part was the bark (*9*).

Nicolas Monardes, a 16th-Century protobotanist, provides a version:

From the new kingdom they bring a bark, which they say is from a tree, which is of great size, which they say, bears heart-shaped leaves, and bears no fruit. This tree has a very solid and hard thick bark, which in this and in color resemble much the bark of the stick they call Guayacán: on the surface it has a thin whitish skin, broken all over it: it has the bark more than a finger thick, solid and heavy, which tasted has remarkable bitterness, like that of the gentian: it has a remarkable taste astriction, with some aromaticity, because at the end of the chewing it breathes a good smell. The Indians have the bark in abundance, and use it in all kinds of xamaras, whether with or without blood. The Spaniards, weary of this disease, on the advice of the Indians, have used this bark and have healed many of them with it. They take of it as much as a small bean made into powders, they are taken in red wine, or in appropriate water, as they have the fever or bad... (*10*).

A.W. Haggis, in his Fundamental Errors in the Early History of *Cinchona*: Part 1 (*3*), said that Antonio de la Calancha (1631) and Sebastiano Bado (1663) mention a “fever tree” from Loja, which has a thick cinnamon-colored bark and a bitter taste (*11**,**12*). Bado wrote, “When two reals of its powder, mixed with wine or another liquid, is ingested, it had the capacity to cure fevers and tertians” (*12*). However, he casts doubt on statements by Joseph de Jussieu, who said that a Jesuit was the first European to be cured of fever by quinquina at Malacatos, and Charles M. de La Condamine, who in 1600 referred to the use of quinquina as a remedy by Europeans living in Lima, because the actual remedy taken in both cited cases could have been *Myroxylon*, which also was used as a febrifuge (*3*). Moreover, Haggis indicates that Schedula Romana (1651), a famous apothecaries guide, was the oldest printed source citing the use of cinchona (*china della febre*) as a remedy for fever (*2*).

In contrast, Francisco Guerra considers Bollo’s letter, cited by Bado in 1663, as the oldest reference to the use of *Cinchona* bark (*13*). The letter states that its febrifuge properties were known and profited by the natives, who prevented the Spaniards from finding out about it (*13*).

In 1663, Caldera reported that both the tree, which was abundant in the province of Quito, and its bark were called *quarango* (*12*). Its medicinal properties were noticed among natives of the Amazon River, who, on their way to a gold mine, swam across an icy river, having shivers afterwards. Drinking powdered *quarango* bark dissolved in hot water provided them immediate relief. When the Jesuits heard about this, they obtained all information from the natives (*14*). The perception that the natives were fully aware of *Cinchona*’s medicinal properties is reinforced by Diego de Herrera’s testimony on his lost manuscript De Cortice Quinae Quinae (1699), cited by La Condamine in 1738 (*10*).

Father Bernabé Cobo noticed that Llano-y-Zapata referred to *Cinchona* as *Lacanna Perida*, a novel name of obscure origin. Cono added that Juan de Vega was the first to use the husk to counteract the fevers of the Viceroy in 1638 (*15*).

According to E. Augusto in 1943, the Spaniards adopted the use of *Cinchona* from Inca medicine (*16*). The first to get cured were Juan López de Cañizares (governor of Quito) and 2 Jesuits from Malacatos (*16*).

In 1963, Alfonso Anda Aguirre attributed *Cinchona* medicinal lore to the native Paltas, from whom the Spaniards took knowledge at Mercadillo (*17*). Regarding Pedro Leiva, chief of the native Paltas, healing the Corregidor de Cañizares (corregidors were political, administrative, and judicial authorities appointed directly by the Spanish Crown to govern a territory or province), Anda Aguirre rejects criticisms pointing out that the chief’s Spanish name undermines the veracity of the story; Spaniards used to name their subjects after themselves, meaning that the native Pedro Leiva could have taken the name of the Spaniard with whom he was socially connected, as was a custom (*17*).

Adding to our knowledge of the role of indigenous people on the discovery of the *Cinchona* tree, in 1995, Eduardo Estrella highlighted 2 important but little-known references (*18*). The first reference was Fernando de la Vega, a merchant and healer born in Loja, who wrote in 1752, at the age of 80 and upon request of Miguel de Santisteban, an extraordinary memoir: “Virtues of the cascarilla made from leaves, buds, bark, powder, and root bark,” considered the first indigenous contribution about the medicinal properties of *Cinchona* (*18*). The second reference was Miguel de Santisteban, a soldier and superintendent of Bogota’s mint, who, by royal order, reported on the situation of cinchona in 1752 and organized its regular shipment to the Royal Apothecary in Madrid. Upon visiting Loja in 1739, he wrote Noticias de la Cascarilla de Loja, an illustrated guide on the tree and its celebrated bark. He also proposed establishing *Cinchona* state-run stores to ensure its quality.

**Figure 1:**
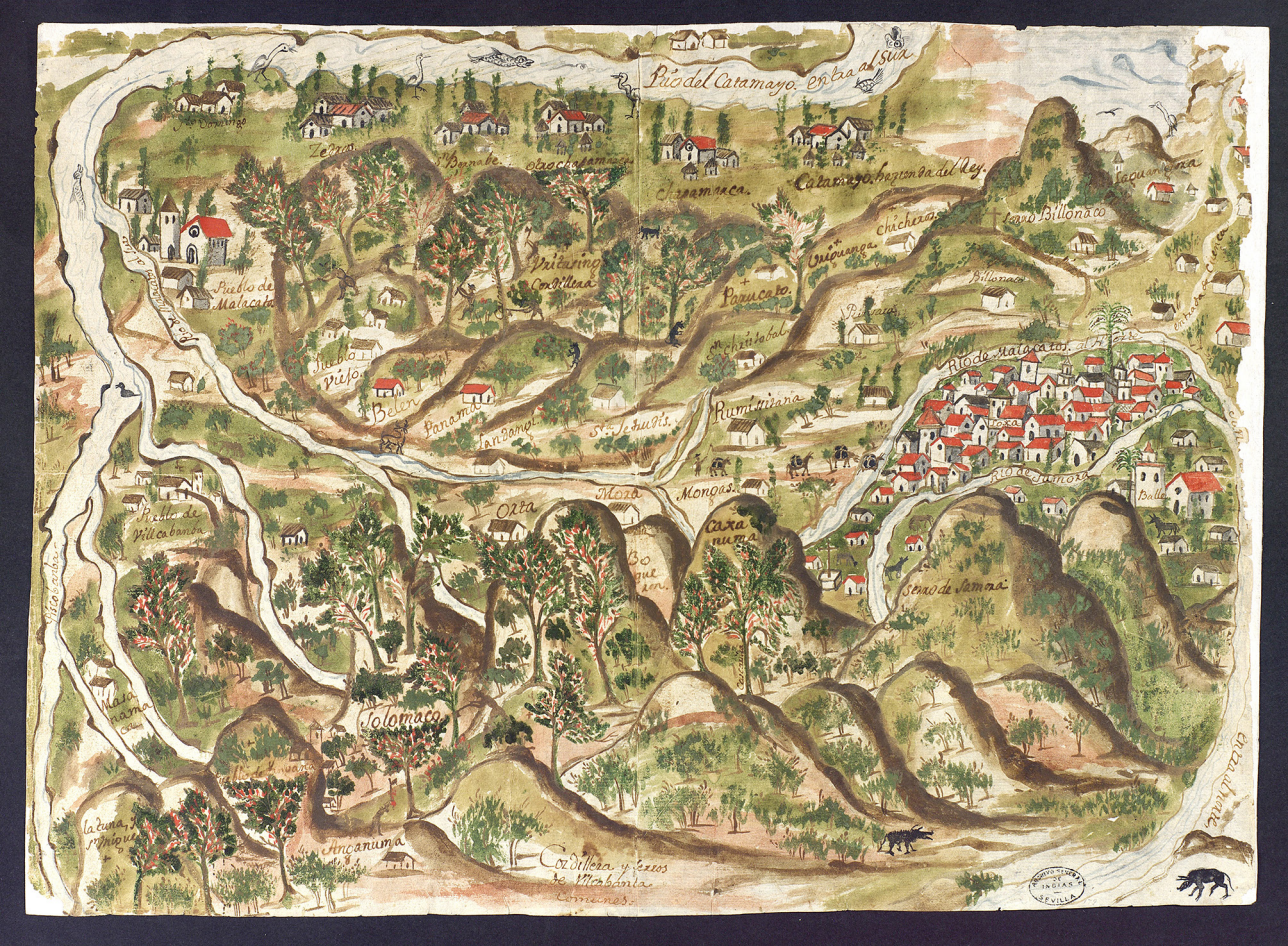
Loja, Peru, and the reserved Vilcabamba mountains where Cinchona trees grow, highlighting trade routes during the Viceroyalty of Peru. Source: General Archive of the Indies, Seville, Spain, 1773. Courtesy of José Carlos Arias.

During the process of extracting the *Cinchona* (*cascarilla*), a trade route was marked out in the mountains of Vilcabamba (Figure 1). The Vilcabamba mountains were populated by *Cinchona* trees during the viceroyalty period, so this geographic region was a center for the use of *Cinchona* for trade and medicine. Jussieu, a botanist of the Catelnau expedition looking for *Cinchona calisaya* during 1843–1845), described how knowledge about the medicinal properties of *Cinchona* was first obtained:

Thanks to the Indians of Malacatos, south of Loja; who suffered intermittent fevers due to the inconsistencies of the climate; they found it necessary to seek a cure and were botanical experts and connoisseurs of the virtues of various types of herbs; after experimenting with various plants they discovered that cinchona bark was the only remedy to cure intermittent fevers, so they called it Yara [tree] Chucchu or Cava [bark] Chucchu [cold]” (*19*).

**Figure 2:**
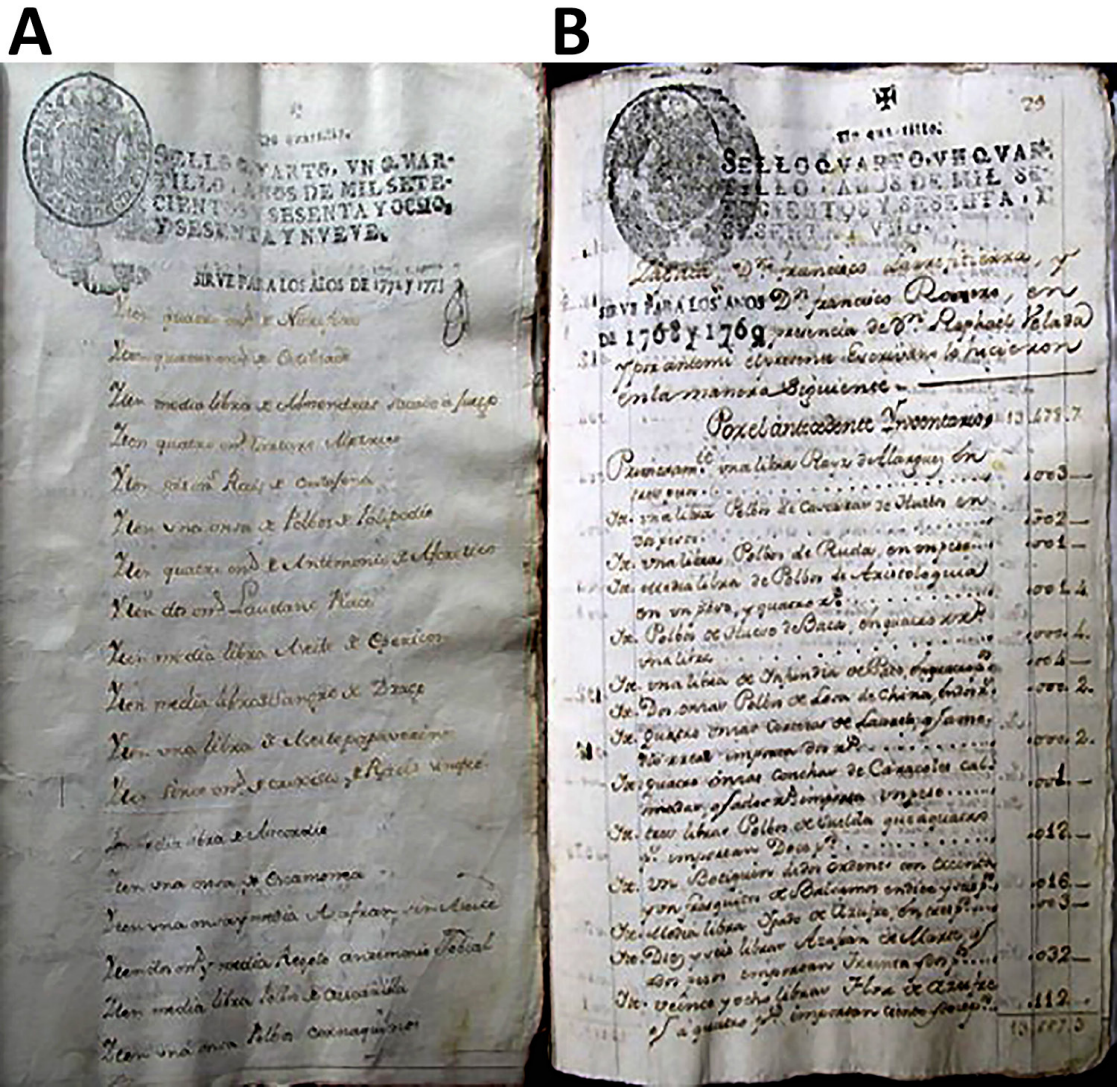
Documents from 1768 in archives of 2 very important locations for the Viceroyalty of Peru (Cuzco and Lima in present-day Peru) referring to “*cascarilla* powder” and “Loja powder” A) “Cascarilla powder” is mentioned in the Archive of the Colegio Ciencias: File 6, book 1, page 112, Cuzco. 1768–1769 (*21*). B) Page of the inventory of the apothecary of Saint Paul's College, where “Loja’s powders of cinchona” is mentioned. Source: Velada R. National Archives of Peru, 1768 (*22*).

José Arias agreed that the natives who discovered *Cinchona* in time immemorial were the Palta, indigenous to Loja (*20*). Early in the 17th Century, Chief Pedro Leiva used it to treat a Jesuit sick with malaria who, in turn, would use the remedy to cure the Corregidor de Cañizares in 1630. Two years later, Juan López would provide it to the wife of the Viceroy of Peru (*20*). Furthermore, previous sources have already demonstrated the presence of *cascarilla* during the Viceroyalty of Peru, both in Cusco and Lima (*21*,*22*) (Figure 2).

*Cinchona* was freely traded until 1752. A year earlier, the Spanish Crown had already intervened in its exploitation, implementing the demarcation of the forests of Loja. Its extraction, selection, collection, and transport were regulated, which had a positive effect on the economy (*20*). Likewise, according to the diary of Don Miguel de Santisteban, exports to Spain, which began around 1640, expanded to include Rome and Paris 10 years later (*23*).

The main sources of the Royal Spanish Apothecary were Loja (1750–1775), followed by Cuenca (1775–1787), after Loja’s forests were depleted (*14*). Despite this event, Loja kept its role as the start point of the export routes, which proceeded from Loja to Catacocha to Celica to Tumbes to Piura and Malacatos to Tumbes to Guayaquil. The main destinations in Spain were the ports of Cádiz and El Ferrol (*24*).

## Set of Historical Contexts as Bases for the Discovery of *Cinchona* Bark (*Cascarilla*) for Treatment of Malaria

### Geographic Conext of *Cinchona* and Precolonial and Colonial Febrile Endemic Diseases in the Region 

Although *Cinchona* originates ancestrally from the central-eastern Andes, historically it has been commercially exploited farther north, in the Andes between Ecuador and Peru. In that region, an important site is Mt. Caxanuma (Loja), described by La Condamine in 1737. Between that year and 1799, he verified the presence of *Cinchona* between Loja and the central Peruvian Amazon. It was most prevalent in Piura and Cajamarca, where the most frequently observed species were *C. pubescens* and *C. officinalis*. Subsequent studies have validated his observations (*25*,*26*). In the described area, on both sides of the mountain range, several important elements converged, including the presence of native communities and their medicinal plants, endemic malaria beginning with the arrival of the Spaniards, and the existence of a north–south trade route for *Cinchona* bark leading to Lima.

Malaria has only been present on the Piura Coast and in the Cajamarca and Amazonas’ jungles since the viceroyalty period. The presence of *Plasmodium* parasites has been documented in a colonial-era mummy from Amazonas (*27*).

*Cinchona* thrives in so-called health axes areas (i.e., Loja, Ecuador–Piura–Cajamarca–Amazonas, and Peru). Those areas are ethnographic regions where communities share concepts of health and principles of folk medicine, such as the “hot–cold” opposition, which is prevalent in Latin America. This method involved administering substances opposite to what they perceived in the patient (e.g., if healers perceived heat in the patient, they would administer a substance they considered cold, and in this way cure the patient) (*28*,*29*). Those areas have the most biodiverse centers worldwide. The relative low altitude of the Andes has enabled the exchange of flora and fauna from both slopes (the eastern rainforest and the western coast) since time immemorial.

In this region, which includes Peru’s Piura–Huancabamba and Cajamarca regions and Ecuador’s Loja and Zamora–Chinchipe regions, Carrion’s disease, a febrile infection caused by *Bartonella* bacteria, has existed since pre-Columbian times, as demonstrated in a Huari mummy. Malaria became established in the valleys, as in the rest of the western tropics, starting in the late 15th Century. Of note, both diseases cause fever and anemia, and their presence overlapped in this region. Therefore, the indigenous people might have used *Cinchona* to treat malaria on the basis of their previous experience treating fevers caused by Carrion’s disease (*30*,*31*). The use of *Cinchona* as febrifuge was documented in a nearly contemporary situation; the Spanish took advantage of the ancestral use of quinine by the Andaquí people (Caquetá, Colombia) to treat the intermittent fevers that afflicted them during their expedition from the Andes to the mouth of the Amazon River during 1541–1561, as described by chroniclers Gaspar de Carvajal, Francisco Vásquez, and Pedrarias de Almesto (*32*–*34*).

Previous studies have demonstrated the presence of *Plasmodium* parasites in Chachapoyas (a remote region in northern Peru) during 1437–1617. Although genomic analyses show similarity to current strains in Peru, those colonial cases undoubtedly represent infections within the context of the European invasion of the Americas (*31*,*35*–*38*). In that case, the antimalarial effect of *Cinchona* might have been discovered because of the use of this plant to treat a febrile illness that causes anemia such as Carrion’s disease.

### Ethnobotanical Knowledge and Cultural and Commercial Exchange with Respect to *Cinchona* Bark (*Cascarilla*)

From the perspective of local ethnomedicine, it is important to understand the use an appropriate treatment prescribes the opposite, as described previously (e.g., cold remedies to draw out the heat and hot remedies to draw out the cold of *Cinchona*) (*28*,*29*). Another principle used by traditional medicine is the sweet–bitter opposition. In ancient times, bitter concoctions, such as *Cinchona* bark solutions, were used to expel evil spirits to which ailments were attributed. Similarly, if an illness was accompanied by sweet fluids (e.g., blood or urine) because of secondary hyperglycemia (e.g., dehydration from fever or adrenergic stress), the condition could be counteracted by administering bitter substances such as *cascarilla*. The native Peruvian–Ecuadorian peoples might have discovered the fever-reducing properties of *Cinchona* within this context. A similar line of reasoning could have been to follow the principle of similarity, using bitter substances to treat fevers associated, for example, with liver disease and other ailments causing bilious vomiting (*39*,*40*). Of note, in Loja, Ecuador, ethnobotany has been used to treat fevers, as demonstrated through a study that documented 25 plants used to treat suspected malaria and other fevers (*39*). Some of those plants belong to the Rubiaceae family, to which the genus *Cinchona* belongs.

The well-documented trade route between northern Peru (Piura) and southern Ecuador (Loja) was the setting where information about the discovery and use of *Cinchona* as an antimalarial spread from the early viceroyalty. The Jesuits played a key role in that process, thanks to the development of their missions, from which they gathered and disseminated all possible knowledge. That role was particularly important for Loja, where Jesuits arrived at the beginning of the colonial period and founded the first secondary school in 1727 (*41*). 

**Figure 3:**
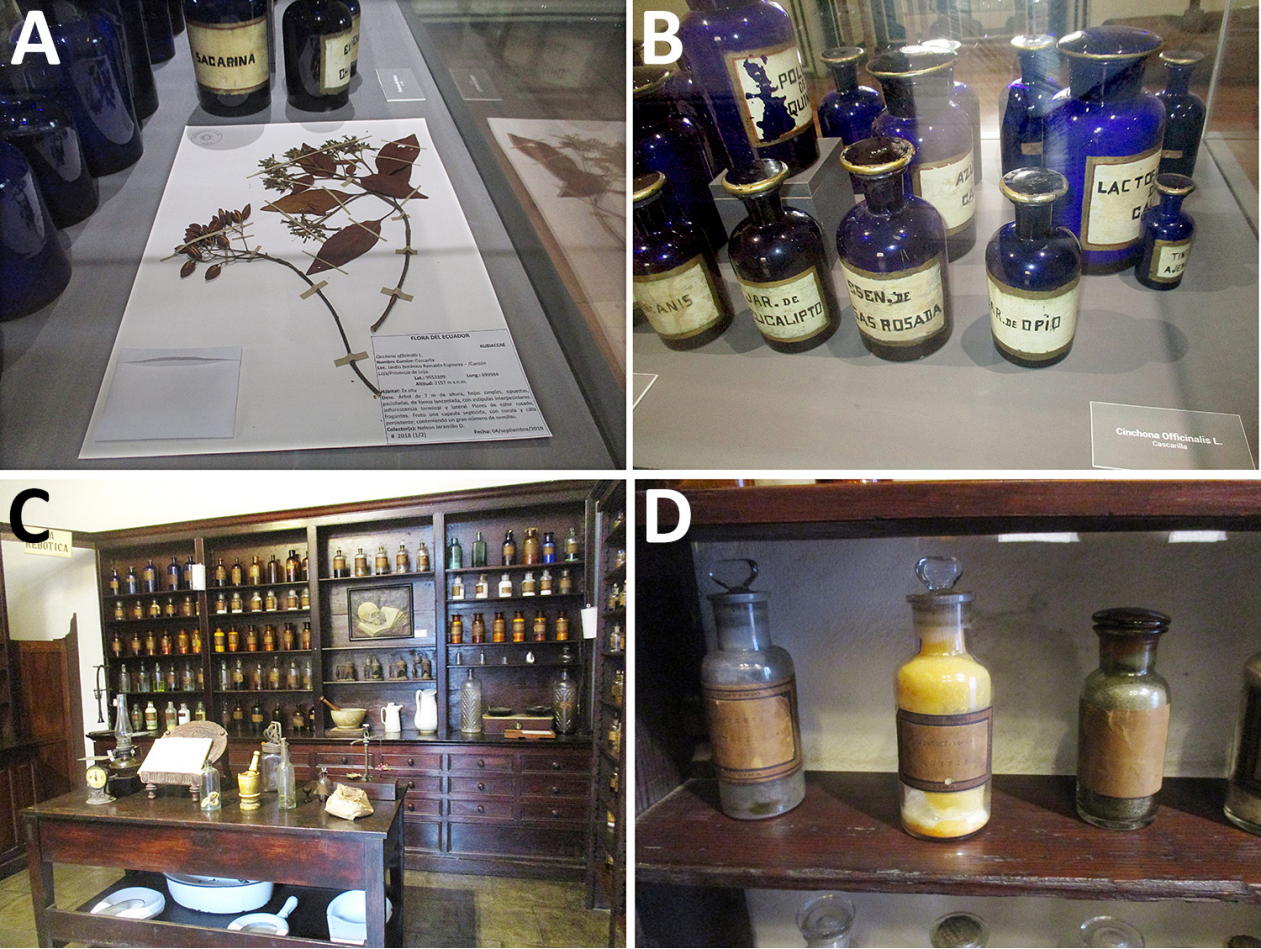
Presence of *Cinchona* in Loja, Ecuador, and Lima, Peru, and its use as powdered medicine. A) Herbarium file of *Cinchona officinalis.* Source: Museum and Cultural Center of Loja, Ecuador. B) Bottle labeled “quinine powder.” Source: Museum and Cultural Center of Loja, Ecuador. C) Pharmacy and medication preparation area. Source: Museum of the Barefoot, Franciscan Order, Lima, Peru. D) Bottle of quinine. Source: Museum of the Barefoot, Franciscan Order, Lima, Peru.

During that time, *Cinchona* was widely traded along various routes that swiftly connected Loja and Cuenca (Ecuador) with Piura and Lima (Peru) and, from there, with Spain. Such trade led to the emergence of a local elite, distinct from that of Lima (*42*). Families were linked through marriage and strengthened ties through business, such as the González de Salazar and Sánchez Navarrete Peninsular-Creole clan of *Cinchona* bark merchants operating between Guayaquil and Lima, through southern Ecuador and northern Peru. The clan established an annual shipment of cinchona bark from Loja to the royal pharmacy in Madrid, which departed from ports in present-day Peru and Ecuador (*43*,*44*). It is important to highlight the recognition of *Cinchona* in the medicinal, commercial, and historical contexts in Lima and Loja during the colonial period and present day (Figure 3)

*Cascarilla* also was obtained, in smaller quantities, in the northern highlands of Piura (Huancabamba and Ayabaca). The continuous trade involved corregidors Matías Joseph de Valdivieso (Piura) and Pedro Javier de Valdivieso (Loja), who also supplied the Royal Pharmacy of Madrid (*45*).

Spaniards such as Diego Vaca de Vega and Jesuit missionaries settled in Maynas (Loreto, Peru), a process during which they became more familiar with local medicinal plants. Vaca de Vega and soldiers from Loja founded the San Francisco de Borja School in 1619 (*46*). That collaboration highlights the relationship between military companies and the Jesuits. They arrived together, having knowledge of the malaria that caused fevers and the traditional medicines used by the native communities based on plants to treat the fevers; therefore, the colonists used the plants for the treatment of fevers and for their trade. 

The intense social and administrative activity along the commercial axis between Cuenca, Loja, Piura, and Lima was dominated by Piura, from where *Cinchona* bark and textiles were also exported through the port of Paita (*47*,*48*). In 1778, *Cinchona* shipment began in Malacatos (Loja), where it was packed and transported to the ports of Tumbes (Peru) or Guayaquil (Ecuador), then on to the port of Callao (Peru), with the final destination being the port of Cádiz in Spain (*21*,*46*).

**Figure 4:**
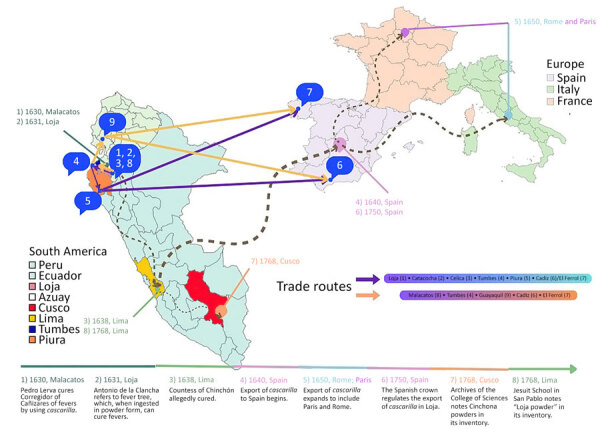
Chronologic and historical milestones of the *Cinchona* bark (also known as *cascarilla* or Loja’s powder).

The documents describing the *Cinchona* trade reveal not only the use and importance of *Cinchona* bark but also its characteristics and the most sought-after and commercially traded varieties. Furthermore, they show the main routes by which this product and its use as an antimalarial spread from Peru to the world, through Europe. They also indicate problems that arose throughout the global distribution of *Cinchona* bark in terms of quality and price. The discovery of *Cinchona* unites aspects of distribution, ethnobotanical, epidemiologic, exchange, and commerce from the pre-Inca era to the Viceroyalty (Figure 4). 

## Discussion

Unfortunately, despite having the *Cinchona* tree on Peru’s national coat of arms, very little information exists about its discovery or early use in Peruvian archives, libraries, and the main headquarters of the Jesuit Order (the Basilica, the minor Convent of San Pedro, and the Apothecary of the Colegio de San Pablo were the main administrative centers and sites of *Cinchona* use by the Jesuit Order during the Viceroyalty of Peru). We do not consider the story about the Countess of Chinchón, which is depicted in a popular novel about the discovery of *Cinchona* as an antimalarial, because it lacks scientific veracity and most researchers now consider this story apocryphal (*2*). Information is limited about the discovery and early use of the *Cinchona* tree. This situation resulted from 3 events: the expulsion of the Jesuits in 1767, during which their documents were relocated or lost; the War of the Pacific during 1879–1884, when the National Library was looted (1881); and the National Library fire in 1943, when even more historical sources were lost.

Within the variety of references to the discovery of the therapeutic use of *Cinchona* bark, a common fact stands out: most authors highlight the indigenous people as the true discoverers. The oldest reference to that fact is from Nicolas Monardes in 1574 (*49*). Haggis, Calancha, and Bado indicate that the natives used *Cinchona* bark as a remedy for fevers (*3*,*11*,*12*). According to Bado, the natives acquired this knowledge late, between the arrival of the Spaniards and the discovery of this remedy by the Europeans (*12*). This observation is supported by a recent evaluation of the genomic variation of *P. vivax* in Latin America; all lineages can be traced to multiple introductions from Europe and Africa that occurred after contact between the natives and the Europeans (*50*).

The discovery of *Cinchona* as an antimalarial occurred because of the confluence of favorable factors that were present since pre-Hispanic times. Those factors include the geographic distribution and endemism of *Cinchona*, Amazonian ethnobotany conducive to treating fevers, endemic febrile diseases such as bartonellosis, and mobility and cultural–commercial exchange.

## Conclusions

Loja was the axis of discovery and a center of supply and export of *Cinchona* in the 17th Century. Viceregal documents abound in this regard, especially highlighting the town of Malacatos and its leader, Chief Pedro Leiva, who raised the *Cinchona* to universal acceptance as a therapeutic intervention for malaria.

Chief Leiva revealed the secret of *Cinchona* as a febrifuge to a Jesuit missionary and, in turn, shared that information with the Corregidor Juan López Cañizares around 1630. From Loja, either by the Jesuits, an administrative, political, and judicial authority such as the Corregidor Juan López Cañizares, or both, the information arrived in Lima, capital of the Viceroyalty of Peru, seat of its political and religious authorities. From Lima, the effectiveness of the bark as an antimalarial expanded exponentially to the world.

Likewise, the Jesuits clearly maintained an early monopoly on the value and use of *Cinchona*. Of this fact there is testimony in different documents (recipe books in the apothecaries of San Pablo and the Jesuits of Santiago).

Both native and European characters played key roles in *Cinchona*’s heritage. The natives, in their long tradition of trial-and-error plant-based medicine, swiftly succeeded when confronted with a newly arrived disease (malaria). The Europeans were responsible for controlling the production and distribution of *Cinchona*, which, in the long run, saved millions of lives.
